# Revisiting Histone Deacetylases in Human Tumorigenesis: The Paradigm of Urothelial Bladder Cancer

**DOI:** 10.3390/ijms20061291

**Published:** 2019-03-14

**Authors:** Aikaterini F. Giannopoulou, Athanassios D. Velentzas, Eumorphia G. Konstantakou, Margaritis Avgeris, Stamatia A. Katarachia, Nikos C. Papandreou, Nikolas I. Kalavros, Vassiliki E. Mpakou, Vassiliki Iconomidou, Ema Anastasiadou, Ioannis K. Kostakis, Issidora S. Papassideri, Gerassimos E. Voutsinas, Andreas Scorilas, Dimitrios J. Stravopodis

**Affiliations:** 1 Section of Cell Biology and Biophysics, Department of Biology, School of Science, National and Kapodistrian University of Athens, 15701 Athens, Greece; Katia_13g@hotmail.com (A.F.G.); tveletz@biol.uoa.gr (A.D.V.); skatarachia@biol.uoa.gr (S.A.K.); npapand@biol.uoa.gr (N.C.P.); vbakou@biol.uoa.gr (V.E.M.); veconom@biol.uoa.gr (V.I.); ipapasid@biol.uoa.gr (I.S.P.); 2 Harvard Medical School, Massachusetts General Hospital Cancer Center, Charlestown, Boston, MA 021004, USA; ekonstantakou@mgh.harvard.edu; 3 Section of Biochemistry and Molecular Biology, Department of Biology, School of Science, National and Kapodistrian University of Athens, 15701 Athens, Greece; margaritis.avgeris@gmail.com (M.A.); ascorilas@biol.uoa.gr (A.S.); 4 Center of Basic Research, Biomedical Research Foundation of the Academy of Athens, 11527 Athens, Greece; nikolas.kalabros@gmail.com (N.I.K.); anastasiadou@bioacademy.gr (E.A.); 5 Division of Pharmaceutical Chemistry, Department of Pharmacy, National and Kapodistrian University of Athens, 15701 Athens, Greece; ikkostakis@pharm.uoa.gr; 6 Laboratory of Molecular Carcinogenesis and Rare Disease Genetics, Institute of Biosciences and Applications, National Center for Scientific Research “Demokritos”, 15310 Athens, Greece; mvoutsin@bio.demokritos.gr

**Keywords:** bladder, cancer, deacetylation, HDAC, inhibitor, therapy

## Abstract

Urinary bladder cancer is a common malignancy, being characterized by substantial patient mortality and management cost. Its high somatic-mutation frequency and molecular heterogeneity usually renders tumors refractory to the applied regimens. Hitherto, methotrexate-vinblastine-adriamycin-cisplatin and gemcitabine-cisplatin represent the backbone of systemic chemotherapy. However, despite the initial chemosensitivity, the majority of treated patients will eventually develop chemoresistance, which severely reduces their survival expectancy. Since chromatin regulation genes are more frequently mutated in muscle-invasive bladder cancer, as compared to other epithelial tumors, targeted therapies against chromatin aberrations in chemoresistant clones may prove beneficial for the disease. “Acetyl-chromatin” homeostasis is regulated by the opposing functions of histone acetyltransferases (HATs) and histone deacetylases (HDACs). The HDAC/SIRT (super-)family contains 18 members, which are divided in five classes, with each family member being differentially expressed in normal urinary bladder tissues. Since a strong association between irregular HDAC expression/activity and tumorigenesis has been previously demonstrated, we herein attempt to review the accumulated published evidences that implicate HDACs/SIRTs as critical regulators in urothelial bladder cancer. Moreover, the most extensively investigated HDAC inhibitors (HDACis) are also analyzed, and the respective clinical trials are also described. Interestingly, it seems that HDACis should be preferably used in drug-combination therapeutic schemes, including radiation.

## 1. Epidemiology—Risk Factors

Cancer of the urinary bladder is a highly prevalent disease, and is closely associated with substantial morbidity, mortality and cost. It is the 9th most frequent malignancy and the 13th most common cause of death, worldwide. In the year 2012, more than 429,000 cases of bladder cancer were diagnosed and more than 165,000 deaths were recorded, globally [1,2]. For 2018, it has been estimated that over 80,000 patients in the United States of America will develop urinary bladder cancer [3,4]. Disease incidence increases with age, with men being affected in a comparatively higher percentage than women, following an approximate 3:1 average ratio. Developed countries are nearly three times more vulnerable to bladder cancer than under-developed countries, with North America and several European, Western Asia and Northern African countries exhibiting the highest age-standardized incidence. Intriguingly, although rates of the disease are higher in white populations, as compared to other ethnicities, survival seems worse for black individuals. Variations in regional mortality could presumably be attributed to the differential access to health care systems, and disparate protocols and facilities utilized for diagnosis and treatment [1,2,5,6,7,8,9,10].

The most common risk factor for bladder cancer is cigarette smoking, with tobacco being estimated to be responsible for almost half of all cases [11,12]. Ambient air pollution, such as the one derived from combustion chemicals, has been also suggested to influence bladder cancer risk, while occupational exposure to certain mutagens in industrial areas has been long associated with development of the disease. Urban living, and diets low in fruit and vegetable are additionally linked to increased incidence of bladder cancer [12,13]. Interestingly, a genetic predisposition to bladder cancer has been previously reported, involving genes that control carcinogen metabolism, such as *N*-acetyltransferase 2 (*NAT2*). Moreover, genome-wide association studies (GWAS) have identified sequence variants that can increase susceptibility to the disease, with alterations in the urea transporter *SLC14A1* gene being characteristic examples [5,14,15,16,17,18,19,20,21,22,23].

## 2. Classification—Genomic Landscapes—Drug Resistance

In North America and Europe, more than 90% of bladder cancers belong to urothelial carcinomas. These tumors are graded according to their cellular properties and staged using the Tumor-Node-Metastasis (TNM) system, which describes their invasion competence (Tis-T4). The low grade, non-muscle-invasive bladder cancer (NMIBC) infrequently acquires invasion features, but can usually recur, following an approximate 5-year survival rate of 90%. In contrast, the high grade, muscle-invasive bladder cancer (MIBC; stage T2 and above) often progresses to metastasis, presenting an unfavorable patient prognosis with a five year survival rate of less than 50%. Since therapy protocols have not advanced for many years, new systemic strategies are necessitated to be promptly developed for the clinical management of the disease [5,24,25,26].

Among the studied malignancies, bladder cancer is presented with one of the highest somatic mutation frequencies, bearing a median value of more than five mutations per megabase. MIBC genome carries a number of gene aberrations that resemble critical ones of other solid tumors. Particularly, loss of function of key tumor suppressors compels cancer cells to evade cell cycle control and apoptosis, and to also deregulate major signaling pathways. Accordingly, the *TP53* and *Rb1* genes are frequently mutated, while regulators of their cognate pathways are also altered, with *MDM2* amplification and *CDKN2A* deletion being two representative paradigms, respectively. Other tumor suppressor genes being inactivated by mutation in MIBC, are the ones encoding the ARID1A, TXNIP, ELF3, NFE2L2, FBXW7, APC, CDKN1A, EP300, TSC1, PTEN, ATM, KMT2D, KLF5 and TSC2 proteins. The MIBC-specific molecular signature also contains mutation-driven activation of several oncogenes, including the *E2F3*, *PIK3CA*, *FGFR3*, *HRAS*, *ERBB3*, *CCND1*, *RXRA*, *HER2*/*ERBB2*, *EGFR*, *FGFR1*, *KRAS* and *AKT1* ones. In contrast to MIBC, NMIBC is genomically stable, with chromosome 9 deletion representing the most common copy number aberration. This leads to *CDKN2A* and *TSC1* loss, and thus subsequent perturbation of the Rb-/p53- and mTOR-dependent pathways, respectively. Additional tumor suppressor genes are also inactivated in NMIBC, containing, among others, the *KDM6A*, *STAG2*, *RBM10*, *CREBBP* and *ERCC2* ones. The NMIBC-specific genomic landscape is also characterized by activating mutations in several key oncogenes (e.g. *TERT*, *FGFR3*, *PIK3CA*, *HRAS*, *KRAS* and *AKT1*), with *TERT* and *FGFR3* exhibiting the highest frequency among all. Interestingly, a powerful, multiplatform analysis has recently identified 5 MIBC subtypes that may stratify response to different treatments. The new subtypes have been characterized as: (a) luminal-papillary (35%; *FGFR3* mutations), (b) luminal-infiltrated (19%; EMT (epithelial to mesenchymal transition) markers), (c) luminal (6%; *KRT20* and *SNX31*), (d) basal-squamous (35%; *CD274/PD-L1* and *CTLA4*) and (e) neuronal (5%; neuroendocrine and neuronal genes). Additional integrative analyses could further refine this novel MIBC subtyping, and notably support the current research for personalized targeted therapies [4,5,6,24,27,28,29,30].

The mortality rate of patients being affected by urothelial carcinoma has not essentially changed over the past 30 years, thus reflecting the limitations of applied therapeutic protocols. Regimens containing platinum-based agents, such as the MVAC (methotrexate-vinblastine-adriamycin-cisplatin) and GC (gemcitabine-cisplatin) ones, represent the backbone of first-line systemic chemotherapy for patients suffering from advanced-stage urothelial carcinoma. Despite the initial chemosensitivity, chemoresistance will eventually develop in the majority of patients, resulting in a median, overall, survival between 13 and 19 months. In the second-line therapy, cisplatin-resistant urothelial carcinomas can be treated with immune-checkpoint inhibitors (e.g. avelumab, atezolizumab and nivolumab), but the clinical benefits remain limited, as more than 60% of the patients die after 12 months. It seems that chemotherapy-resistant urothelial carcinoma has undergone a dynamic and extensive clonal-evolution program, with critical changes in its mutational landscape being accumulated during and after chemotherapy. Hence, capture and quantification of heterogeneity in a urothelial tumor’s molecular architecture, will enable the early identification and targeting of key molecular events occurring during a tumor’s evolutionary trajectory, favoring the emergence of chemotherapy-sensitive and immune-responsive clones. Given that the *ARID1A*, *CREBBP*, *EP300*, *KDM6A* and *KMT2D* genes encode chromatin modifiers, epigenetic alterations may hold a central position in the therapy-resistance map of urothelial carcinoma. In accordance, chromatin regulation genes are more frequently mutated in MIBC than in other epithelial cancers examined so far, thus suggesting the strong potential of targeted therapy for chromatin abnormalities in chemoresistant clones [5,6,24,25,29,31,32,33,34,35,36,37,38,39,40].

## 3. Chromatin Remodeling—Acetylation versus Deacetylation—HATs

**Figure 1:**
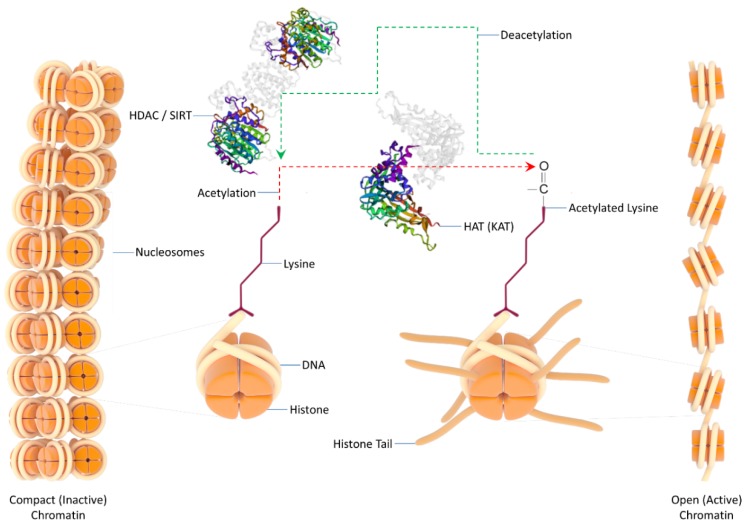
Graphic presentation of the acetylation and deacetylation processes. They can be implemented by the respective functions of HAT and HDAC enzymes, which regulate (among others) the acetyl-lysine load of nucleosomal histones, thus controlling the compact (transcriptionally inactive) or open (transcriptionally active) architecture of nuclear chromatin. HAT(1): PDB 2p0w; HDAC(1): PDB 4bkx [47,48].

Eukaryotic DNA is tightly wrapped around a cluster of eight proteins called core histones, in order to form nucleosomes, the fundamental units of chromatin. Several post-translational modifications of histones, such as ubiquitylation, phosphorylation, methylation and acetylation, are critical components of the epigenome (which literally means “above” genome), with “acetyl” marks, besides being classified among the most abundant modifications, also providing a large collection of druggable proteins that control the “acetyl-genome” dynamics and functions. Acetylation neutralizes the positive charge of lysine, thus weakening the charge-dependent interactions between nucleosomal DNA and histones. As a result, chromatin becomes structurally more relaxed, and DNA accessibility to the transcription machinery is increased. In general, hyper-acetylated histones are associated with transcriptionally active genes, whereas hypo-acetylated histones are mechanistically linked to transcriptionally repressed genes (Figure 1). Interestingly, acetylation of lysine 16 on Histone 4 (H4K16) seems to play an important role in chromatin folding regulation and transition from heterochromatin (high compaction) to euchromatin (low compaction) form. Histone acetylation is a highly dynamic process being regulated by two enzyme family members, operating in an opposite fashion: The histone acetyltransferases (HATs) and the histone deacetylases (HDACs). HATs use acetyl-CoA as co-factor to catalyze the transfer of “acetyl” molecule to the ε-amino group of lysine side chain on histone N-terminal tails. In contrast, HDACs oppose the HAT effects and reverse lysine acetylation status, to restore the lost positive charge and stabilize local chromatin in a compact architecture (Figure 1) [41,42,43,44,45,46].

There have been identified nine mammalian nuclear HATs, with structurally defined acetyltransferase domains and histone lysine-specific acetylation activities, mainly organized in pairs: GCN5/KAT2A and PCAF/KAT2B (pair I); CREBBP (CBP)/KAT3A and EP300/KAT3B (pair II); MOZ (MYST3)/KAT6A and MOZ2 (MYST4)/KAT6B (pair III); TIP60/KAT5 and MOF (MYST1)/KAT8 (pair IV); and HBO1 (MYST2)/KAT7 (KAT: lysine (K) acetyltransferase; alternative nomenclature) [49]. For example, KAT3A and B show strong preference for H3K18 and 27, KAT2A and B for H3K9, and KAT8 for H4K16. Notably, KAT8 is widely distributed in the genome, and seems to be recruited at gene loci for their transcriptional activation. Similarly, KAT3A and B can occupy gene enhancers, via a transcription factor-mediated recruitment, to induce H3K27 acetylation, and thus open chromatin and activate transcription. Altogether, to locally remodel chromatin from an inactive to an active state, covalent marks (acetyl-lysines) are required to be established by molecular “writers” (HATs), while the reverse process is controlled by the actions of molecular “erasers” (HDACs) that alleviate (epi)genome from the chemical load (acetylation). In between “writing (W)” and “erasing (E)”, there is also a step of (“acetyl”) “reading (R)”, during which chromatin marks (acetyl-lysines) can be recognized by proteins carrying a region of approximately 110 amino acids called bromodomain. The dynamic interplay among these processes (“WRE”) modifies chromatin architecture, reprograms gene expression and provides cellular identity [41,42,44,45,50,51,52,53,54,55,56,57,58,59,60,61,62].

## 4. HDACs: Taxonomy—Activity

Mammalian genomes contain 11 HDAC family members, which are divided into four “phylogenetic” classes: HDAC1, 2, 3 and 8 (class I); HDAC4, 5, 7 and 9 (class IIa); HDAC6 and 10 (class IIb); and HDAC11 (class IV). Another group of deacetylases, the SIRTUINS (SIRTs), compose the class III (SIRT1-7) of the deacetylase super-family. Enzymes that belong to class I, II (a and b) and IV require a divalent metal ion (Zn^2+^) for catalysis, while SIRTs function as NAD^+^-dependent enzymes, carrying protein deacetylase and ADP-ribosylase activities, as well [42,45,63,64,65,66,67,68].

***Class I HDACs***: Class I members are ubiquitously expressed, with the exception of HDAC8 that follows a muscle-specific pattern of expression. HDAC1 and 2 are mainly localized in the nucleus, while HDAC3 shuttles between cytoplasm and nucleus. HDAC1 and 2 serve as critical components of the SIN3A, NuRD and CoREST transcriptional complexes that are being recruited to promoters by DNA binding proteins in order to induce gene-specific transcriptional silencing. Interestingly, HDAC3 is detected in distinct complexes, such as in N-CoR-SMRT, whereas no complex has been hitherto identified for HDAC8. All four enzymes are essentially implicated in cell proliferation and survival. For example, HDAC1 and 2 can normally repress p21^WAF1/CIP1^ and p57^KIP2^ (CDK inhibitors) expression, thus regulating the G1- to S-phase transition of the cell cycle [63,64,65,66,67,69,70,71,72].

***Class IIa HDACs***: Class IIa members are presented with rather restricted and tissue-specific expression profiles. HDAC5 and 9 are enriched in brain, heart and muscles, HDAC4 in skeleton growth plates and brain, and HDAC7 in thymocytes and endothelial cells. In response to their phosphorylation by protein kinases (e.g. CaMK and PKD), they bind to the 14-3-3 anchor protein(s) and shuttle from the nucleus to cytoplasm. Hence, MEF2 transcription factor is dissociated from the HDAC-dependent complex and subsequently interacts with the HAT EP300, converting itself from a transcriptional repressor to a transcriptional activator. Class IIa HDAC deacetylase functions are minimal, and their transcription repressive activities partly derive from the recruitment of class I HDACs (e.g. HDAC3), and also from the interaction with the HP1 and CTBP transcriptional repressors. Interestingly, a substitution of a critical tyrosine to histidine in the catalytic pocket of class IIa HDACs seems to account for their significantly reduced catalytic activities (as compared to the class I HDAC respective ones). Most likely, the intrinsic catalytic domains of class IIa HDACs are not required for the repression process to occur [63,64,65,66,67,73,74,75,76,77,78,79,80,81,82,83,84].

***Class IIb HDACs***: Despite our poor knowledge regarding its function, HDAC10 seems able to promote angiogenesis in endothelial cells. However, it is the other class IIb member, HDAC6, that is considered as the major cytoplasmic deacetylase in mammalian cells. HDAC6 differs from the other HDACs, since it carries 2 deacetylase domains and a C-terminal Zn^2+^-finger. Among the targets being directly deacetylated by HDAC6, there have been recognized two cytoskeletal proteins, the α-Tubulin and Cortactin, which play important roles in cell mitosis and migration, respectively. HSP90 and IFNαR, also, represent genuine targets of the HDAC6 enzyme for direct deacetylation. In addition, HDAC6, together with HDAC9 and SIRT1, can mediate the FOXP3 deacetylation, thus controlling T(reg) cell development and immunosuppressive activity [45,63,64,65,66,67,85,86,87,88,89,90,91,92,93].

***Class IV HDACs***: HDAC11 expression is enriched in the brain, heart, muscles, kidney and testis. It contains a central deacetylase domain flanked by small N- and C-terminal extensions. Although it is predominantly localized in the nucleus, HDAC11 co-precipitates with the cytoplasmic HDAC6. Notwithstanding the development-dependent and region-specific expression profiles having been observed in mouse brain, its function remains poorly understood. Interestingly, HDAC11 has been critically implicated in the regulation (suppression) of IL-10 expression in antigen presenting cells. Moreover, its key role in the control of metabolism and obesity has been recently reported [63,64,65,67,94,95,96,97].

***Class III HDACs***: SIRT1 is predominantly nuclear, and is expressed in the majority of mammalian tissues. It is activated upon nutrient deprivation, and, besides histones, is able to deacetylase transcription factors (e.g. p53) and signaling proteins (β-Catenin), thereby altering their activities. SIRT1 is associated with an increase in lifespan and memory, and presents beneficial effects in metabolic syndrome, neurodegeneration and cancer [45,64,66,67,68,98,99,100]. SIRT2 acts in the cytoplasm and deacetylases α-Tubulin, thus playing a redundant role with HDAC6 in microtubule homeostasis. SIRT2 also deacetylases and regulates the APC/C complex that is required for cell-cycle progression and exit from mitosis. It has been also reported to critically contribute, via a G6PD deacetylation and activation process, to glucose homeostasis [64,67,68,101,102,103,104]. SIRT3 is a mitochondrial protein that shares the highest structural similarity with SIRT2. It is considered as the major mitochondrial deacetylase, and can be upregulated during calorie restriction or fasting. ACS2 has been shown to serve as one of its important substrates, even though SIRT3 is suggested to play a broad role in the modulation of global mitochondrial lysine acetylation. Notably, it acts as a pivotal regulator of metabolism and oxidative stress (drives Krebs cycle, fatty acid oxidation and urea cycle) [64,67,68,105,106,107,108,109,110]. SIRT4, a mitochondrial enzyme, presents a weak deacetylase function, but possesses a strong ADP-ribosyltransferase activity. In contrast to other SIRT family members that are activated upon energy-deprivation conditions, SIRT4 expression increases at higher levels under nutrient-enriched settings. It can inhibit glutamine catabolism via ADP-ribosylation and repression of GLUD1 (GDH/GLUD), a rate-limiting enzyme in glutamine catabolism [67,68,111,112,113]. SIRT5 predominantly resides in the mitochondrial matrix, and, although it carries an extremely weak deacetylase activity, it can efficiently catalyze the removal of glutaryl-, malonyl- and succinyl-groups from modified (“acylated”) lysines on target proteins. SIRT5 regulates a number of protein substrates critically implicated, among others, in glycolysis, fatty acid oxidation, TCA cycle, ketone body formation, nitrogenous waste management and ROS detoxification. It also critically contributes to stress response and cardiac physiology [64,67,114,115,116,117,118]. SIRT6 is a nuclear protein possessing a deacetylase activity against various substrates, while it is critically involved in the regulation of telomeric chromatin during the S-phase of cell cycle. SIRT6 has proved able to suppress multiple HIF1α target genes known to control glycolysis, and to also co-repress MYC transcriptional activity that functions as an essential modulator of ribosome homeostasis. It is tightly associated with chromatin, and regulates gene expression, genomic stability, glucose metabolism and inflammation [64,67,68,119,120,121,122,123]. SIRT7 also resides in the nucleus, and carries a deacetylase function against several targets involved in the regulation of transcription and metabolism, with p53 protein being a characteristic example. Interestingly, upon endoplasmic reticulum stress, MYC recruits SIRT7 onto promoters of ribosomal genes, co-repressing their transcriptional expression. In accordance, SIRT7 loss mitigates MYC repression, and leads to metabolic deregulation. SIRT7 is enriched in the nucleolus, and facilitates RNA polymerase I activation to control ribosomal RNA expression. Furthermore, hepatic SIRT7 seems to regulate lipid metabolism in the liver in an ubiquitin/proteasome-dependent manner [67,68,124,125,126,127,128].

**Figure 2:**
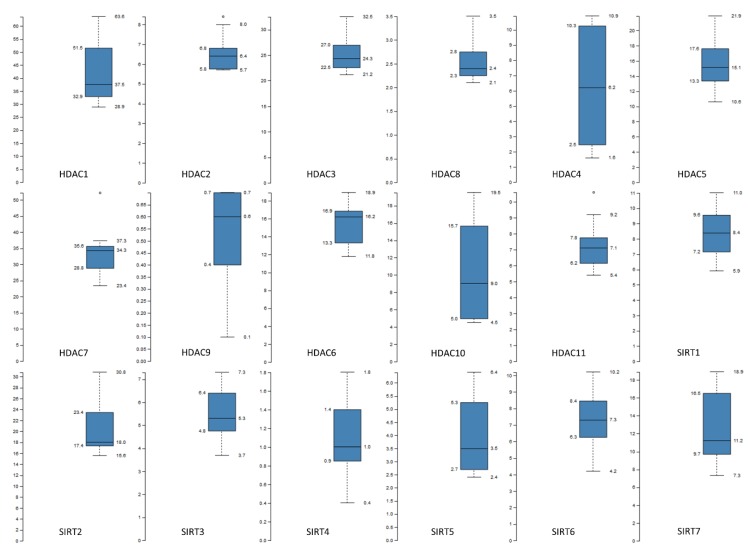
Quantification of *HDAC* expression levels in normal human urinary bladder tissues (n = 11), via employment of an RNA sequencing approach, with the median RPKM (reads per kilo base per million mapped reads) value for each member of the family being indicated on the right side of the respective horizontal line [129].

Exploration of The Human Protein Atlas [129] and especially the part of tissue-restricted proteome expression (Tissue Atlas) [130,131] can unveil the urinary bladder-specific map of all HDAC family member expression landscapes (Figure 2). Based on an integrated “omics” approach that involves immunohistochemical staining and deep RNA sequencing, the expression levels of each HDAC deacetylase have been measured and quantified in normal human bladder tissues. Remarkably, there has been detected a big range of *HDAC* expression among the 18 family members (as evidenced by an RNA sequencing strategy), with *HDAC1* and nine being presented with the highest (37.5) and lowest (0.6) median RPKM (reads per kilo base per million mapped reads) values, respectively (Figure 2). Hence, a significant increase in HDAC9 and/or a notable reduction in HDAC1 cellular contents, may both lead to tissue pathologies and especially to urothelial bladder malignancies. Most likely, the significantly different expression profiles of HDAC enzymes, directly reflect the differential contribution of their respective target genes and protein substrates to urinary bladder development, physiology and disease, including NMIBCs and MIBCs.

## 5. HDACs: Deregulation—Oncogenesis

### 5.1. Aberrant Recruitment

The strong association of aberrant HDAC activity with tumorigenesis has been well demonstrated in acute promyelocytic leukemia (APL). APL is characterized by leukemic cell arrest at the promyelocytic stage of myeloid differentiation, mechanistically caused by the production of fusion proteins, with PML-RARα being detected in the majority of examined cases. Normally, RARα acts as a transcription factor, which can heterodimerize with RXR and bind to DNA, while in the absence of retinoic acid (its cognate ligand) it can recruit HDAC-containing complexes to transcriptionally repress genes. Retinoic acid induces a conformational switch and release of HDAC-dependent repressing complexes, promoting the interaction of RARα with transcriptional co-activators, including HATs, to activate genes. In APL disease, RARα fusion proteins retain the ability to bind to DNA of target promoters, but cannot respond to physiological doses of retinoic acid and, thus, HDAC-carrying complexes remain associated with retinoic acid target genes, causing their constitutive transcriptional silencing. Strikingly, the repression effects are further reinforced by fusion protein oligomerization, that leads to an increased stoichiometric interaction with HDAC-containing complexes and the recruitment of additional chromatin modifiers, such as DNA and histone methyl-transferases. Similarly, the AML1-ETO oncogenic fusion protein identified in t(8;21) AML (acute myeloid leukemia) patients has the capacity to recruit the HDAC1, 2 and/or 3 family members, thereby repressing AML1 target gene expression, which ultimately results in the prevention of myeloid differentiation and induction of cellular transformation [42,66,132,133,134,135,136,137,138,139].

### 5.2. Aberrant Signaling

A molecular link between mitogenic signaling and altered HDAC activity is provided by the RAS-MAPK/ERK-HDAC4 axis. Expression of oncogenic RAS (or, constitutively active ERK1 serine/threonine kinase) causes a significant increase in the number of cells that contain HDAC4 in the nucleus, a compartmentalization process that is associated with kinase activity. In the same direction, an ERK kinase-mediated phosphorylation of HDAC6 is able to promote cell migration via a tubulin deacetylation mechanism. Threonine 1031 and serine 1035 of HDAC6 can be phosphorylated by ERK1 in vitro, while serine 1035 is phosphorylated in response to activation of the EGFR-RAS-ERK signaling pathway in vivo. Therefore, the RAS-ERK-HDAC4/6 axis may critically contribute to cancer initiation, progression and metastasis. Interestingly, phosphorylation of HDAC1 at the critical serine 421 by the NLK serine/threonine kinase (an atypical MAPK family member) results in reduced β-Catenin/LEF1 promoter activation, thus indicating the proficiency of NLK-HDAC1 axis to downregulate WNT signaling, which is vital for the prevention of aberrant proliferation of non-transformed, primary, fibroblast cells. Likewise, the c-ABL (ABL1/ABL) protein kinase can induce tyrosine phosphorylation of HDAC2, affecting (upregulating) both its stability and repression activity in neurons. Given the major importance of the oncogenic fusion protein BCR-ABL (carries aberrant tyrosine kinase activity) in t(9;22) CML (chronic myeloid leukemia) patients, a pivotal role of aberrant HDAC2 function in CML pathogenesis is herein suggested to occur. Remarkably, the JNK kinase (a typical MAPK family member)-dependent phosphorylation of HDAC3 is associated with a five times upregulation of its enzyme activity and three to 16 times increase in HDAC inhibitor selectivity for HDAC3 in triple-negative breast cancer cells, as compared to luminal subtypes. These findings highlight how HDAC phosphorylation affects HDAC inhibitor binding and selectivity, and underscore the significance of JNK-HDAC3 axis in -breast- cancer clinical management and therapy [66,140,141,142,143,144,145,146].

### 5.3. Aberrant Expression

Besides their aberrant (oncogenic) recruitment and signaling, individual HDACs are also subjected to altered expression in a tumor-specific fashion. For example, HDAC1 is over-expressed in breast, gastric and prostate carcinomas, while elevated HDAC2 levels have been detected in cervical and colorectal cancers [65,135,147,148,149,150,151]. In addition, strong HDAC8 expression is associated with neuroblastoma tumorigenesis, while HDAC5 and 9 are significantly upregulated in high- versus low-risk medulloblastoma [135,152,153]. Similarly, acute lymphoblastic leukemia (ALL) is characterized by increased contents of HDAC3 and 7, as compared to a normal bone marrow collection [135,154]. However, HDAC over-expression does not always serve as a negative prognostic biomarker for the disease. Indeed, better prognosis in patients carrying estrogen receptor-positive breast cancer is correlated with elevated HDAC6 levels, thus indicating that acetyl-lysine load may not necessarily ensure clinical sensitivity to chemotherapeutic drugs [135,155]. It must be the tumor-dependent mutational landscape, and its specific cross-talk with a certain HDAC family member that compel each deacetylase to act either as tumor inducer or as tumor suppressor. Interestingly, SIRT1 seems to play a tumor suppressor role, since it is downregulated in several types of cancer, including prostate cancer, ovarian cancer, liver cancer and glioblastoma, with its over-expression in mice delaying the development of KRAS^G12V^-driven lung adenocarcinomas [68,156,157]. In the same direction, liver-specific knockout of *HDAC3* gene leads to hepatocellular carcinoma, while reduced HDAC1/2 enzyme activity causes neoplastic transformation of immature T cells concomitant with increased MYC levels [134,158,159,160]. Intriguingly, a MYC-dependent expression of HDAC2 enzyme can be induced upon loss of the APC tumor suppressor in colorectal tumorigenesis, while crossing of HDAC2 mutant with tumor-prone APC^min^ mice is able to produce significantly lower, as compared to APC^min^ mice (with unimpaired HDAC2), tumor rates [134,151,161], strongly indicating the prominent contribution of specific mutational signatures and tumor settings to the modulation of HDAC2 oncogenic, or onco-repressive function. Most likely, a similar mechanistic principle can be applied to all HDAC family members. In accordance, HDAC1 has remarkably proved to play a dual role in tumorigenesis (e.g. APL); onco-suppressive in early stages and oncogenic in established tumors [162].

## 6. “HDACing” Bladder Cancer

Class I HDACs are the most extensively characterized members of the family in urothelial bladder cancers. The ability of, siRNA-mediated, HDAC1 and 2 double knockout to cause significant impairment in cell proliferation and clonogenic growth, and to also induce apoptotic death of urothelial carcinoma cell lines strongly indicate the critical contribution of class I HDAC member activities to disease initiation and progression [163]. In accordance, in a small-scale study, *HDAC1* mRNA was expressed at notably higher levels in urinary bladder cancer versus normal tissues, while the cognate protein was intensively compartmentalized in the tumorigenic, but not control, nuclei [164,165]. A similar scale collection of patients could also reveal upregulated *HDAC1* mRNA levels in the malignant state, as compared to the healthy one [165,166]. Furthermore, examining a larger cohort of urothelial bladder cancer patients, 40%, 42% and 59% of the tumors were presented with high levels of HDAC1, 2 and 3 protein expression, respectively, with HDAC1 and 2 family members being associated with higher tumor grades, and HDAC1 being tied in with a trend towards poorer prognosis [165,167]. Interestingly, in a similar size patient collection, a statistically significant HDAC1, 2, 3 and 8 over-expression was observed for bladder tumors, as compared to normal mucosas, with HDAC1 and 3 being downregulated in deeply invasive and advanced tumors, and HDAC3 being increased in patients characterized by improved survival [165,168]. The utilization of HDAC1 as diagnostic biomarker and druggable target for bladder cancer is further reinforced by the prominent elevation of its mRNA contents in clinically cancerous tissue biopsies versus healthy tissue biopsies, recently reported in a study of 88 patients [169]. In the same direction, a small number of samples derived from human bladder cancers and adjacent non-malignant tissues were analyzed via a cDNA microarray technology, unveiling the differential expression and upregulation of *HDAC2* gene in metastatic advanced Egyptian bladder cancer [170]. Likewise, several urothelial cancer cell lines (e.g. RT4, RT112 and T24) seem to carry increased levels of *HDAC2* mRNA, as compared to normal uroepithelial cells obtained from healthy human ureters. Notably, *HDAC8* mRNA was also presented with elevated levels in a distinct collection of cell lines, while *HDAC8* gene proved to follow an upregulated pattern of activity in tumor versus control specimens [165,171]. However, not any significant association of *HDAC2* and 8 transcript levels with malignancy grades or stages of the disease could be observed [168]. Intriguingly, the siRNA-mediated *HDAC8* knockout could not impair the viability of urothelial cancer cell lines in a therapeutically useful manner, thus questioning its capacity to serve as a promising and powerful drug target for bladder cancer [172]. Regarding the HDAC3 family member, its expression profiles seem rather varying among the performed studies (including cells and biopsies), therefore necessitating the development of more specific and efficient detection tools, such as novel antibodies with improved abilities for immunohistochemical applications [165,167,168,171]. Nevertheless, an urothelial tumor cell-specific cross-talk between, still elusive, oncogenic signals and *HDAC3* gene transcription cannot be excluded. The bladder cancer cell line-dependent regulation of *HDAC1* gene (and its cognate protein product) [163,165,171] seems to further support the functional interactions of certain tumorigenic determinants with HDAC transcription machineries, in specific settings of malignancy.

All HDAC expression landscapes have been molecularly mapped in a number of urothelial bladder cancer cell lines being typified by different malignancy grades and stages [165,171]. Despite their moderately varying expression profiles, the class IIa HDAC family members 4, 5 and 7 were presented with a notable tendency of transcriptional downregulation in cancerous environments, while *HDAC9* mRNA was shown with upregulated levels in 5/18 of the examined cell lines, indicating, again, the importance of specific oncogenic components to the control of each HDAC expression and activity. Accordingly, but unlike HDAC2 and 8, protein levels of HDAC4 and 7 were found generally reduced in malignant versus normal uro(epi)thelial cells. However, no statistically significant difference of *HDAC9* mRNA contents was observed in 24 bladder cancer specimens when compared to 12 control (morphologically normal) tissues. The mechanistic complexity of HDAC9-mediated deacetylation of bladder cancer cell substrates is further underlined by patterns of decreased *HDAC9* expression being derived from publicly available microarray data. In contrast, using the same platform, *HDAC2*, 3 and 8 were presented with a proclivity towards tumor-dependent augmented transcription, thus indicating the differential contribution of each HDAC enzyme to bladder cancer development [165,171]. With respect to HDAC4, and using bladder cancer tissue arrays coupled to an immunohistochemical protocol, the frequency of HDAC4-positive tissues (but not of HDAC2-positive ones) was significantly higher in the tumor than normal specimens, with the HDAC4 protein levels (in contrast to HDAC1 and 2 ones) being also elevated in a certain collection of bladder cancer cell lines [165,173]. It seems that different HDAC family members can be deregulated by different oncogenic cues, indicating the molecular heterogeneity of “acetyl-signatures” in urothelial tumors, a phenomenon that may likely drive clonal evolution, and cause drug resistance and therapy failure.

Hitherto, only a few studies investigating the expression patterns of class IIb HDAC enzymes in bladder cancer have been published. It has been shown that a significant number of bladder cancer cell lines are characterized by downregulated *HDAC6* mRNA levels, although the cognate protein is presented with a notably varying expression among them [165,171,174]. Intriguingly, in one cohort of patients, a moderate *HDAC6* over-expression was observed in urothelial cancer versus control (normal) tissues, while in 1/6 (publicly available) microarray data reports, elevated transcriptional activity of the gene was also detected. However, these findings could not be confirmed in the remaining five microarray studies and in a different cohort of bladder cancer patients, as well [165,171,174]. Importantly, the siRNA-mediated reduction of HDAC6 protein contents in bladder cancer cells could not induce apoptotic death and diminished viability [174]. Altogether, it seems that bladder carcinomas may not critically depend on HDAC6 expression for cell survival and growth. Nevertheless, an HDAC6-driven deacetylation process may still operate in selected settings of urothelial tumors carrying specific oncogenic signatures. Regarding *HDAC10*, the second gene member of this class, it has been previously presented with varying mRNA expression profiles in a selected collection of bladder cancer cell lines, although a tendency towards its moderate upregulation in carcinoma versus normal urothelial tissues has been also observed [165,171,174].

The class IV family member HDAC11 represents a poorly investigated enzyme in urothelial bladder cancer. Again, examination of several bladder cancer cell lines, being typified by distinct malignancy grades and stages, revealed varying patterns of *HDAC11* gene activity, with 10/18 lines carrying decreased, 5/18 unchanged and 3/18 elevated mRNA levels [165,171]. Similarly to other histone deacetylases, *HDAC11* transcriptional expression may be critically controlled by specific oncogenic networks differentially operating in each bladder cancer cell line examined. Since its depletion seems sufficient to cause death in neuroblastoma, breast, ovarian, prostate and colon cancer cell lines [175,176], HDAC11 could likely function as a major regulator of solid tumors survival and progression, thus holding strong promise to serve as a druggable determinant for bladder cancer targeted therapy.

To elucidate the role of SIRT1, a key member of class III HDAC enzyme family, in urothelial tumorigenesis, its levels of expression in clinical cancer samples were appropriately measured and quantified. In one report, using publicly available data sets, its expression levels were downregulated in bladder carcinomas versus normal tissues, suggesting a tumor suppressor function for the disease [68,156]. However, in another study, a notable *SIRT1* upregulation in bladder cancer (as compared to matched control) tissues was observed, thus indicating its oncogenic role in urothelial malignancies. In accordance, *SIRT1* knockout has proved able to suppress bladder cancer cell proliferation, viability and migration, and also reduce the EMT program potency [177]. It seems that depending on the molecular signature of a given urothelial bladder tumor and its micro-environment, SIRT1 deacetylase may act either as a repressor or as an inducer of the disease. In the same direction, a specific siRNA-mediated targeting of *SIRT2* mRNA, combined with a tubacin-induced inhibition of HDAC6 family member, resulted in significant suppression of bladder cancer cell migration and invasion capacities, strongly supporting their (SIRT2 and HDAC6) cooperative actions and tumor promoting roles in urothelial malignancies [178]. Similarly, the ability of SIRT3 to partially abrogate p53 activity to enact growth arrest and senescence in a bladder carcinoma cell system [179] points out the oncogenic properties of SIRT3 in urothelial tumors. In contrast, the SIRT4 mitochondrial family member can repress tumor formation, with its mRNA expression levels being notably reduced (relative to normal tissues) in a number of malignancies, including bladder cancer [112,113], therefore rendering SIRT4 a promising druggable biomarker for the disease. Likewise, *SIRT6* also acts as a tumor suppressor gene, since its over-expression can inhibit proliferation of bladder cancer cells, while its expression (as detected by an immunohistochemical approach) decreases (in radical cystectomy samples) with progression of urothelial bladder cancer from T2 to T4 stage [180]. On the other hand, *SIRT7* has been previously presented with an upregulated transcriptional activity in bladder carcinomas versus matched normal tissues, with the high-grade and stage tumors being characterized by elevated *SIRT7* expression levels, as compared to the low-grade and stage ones [181], thus indicating the enzyme’s critical oncogenic role in urothelial bladder tumorigenesis. The siRNA-mediated *SIRT7* downregulation that causes increased apoptosis, and reduced proliferation and motility of bladder cancer cells [181] further supports the tumor promoting capacity of SIRT7 deacetylase for the disease.

**Figure 3:**
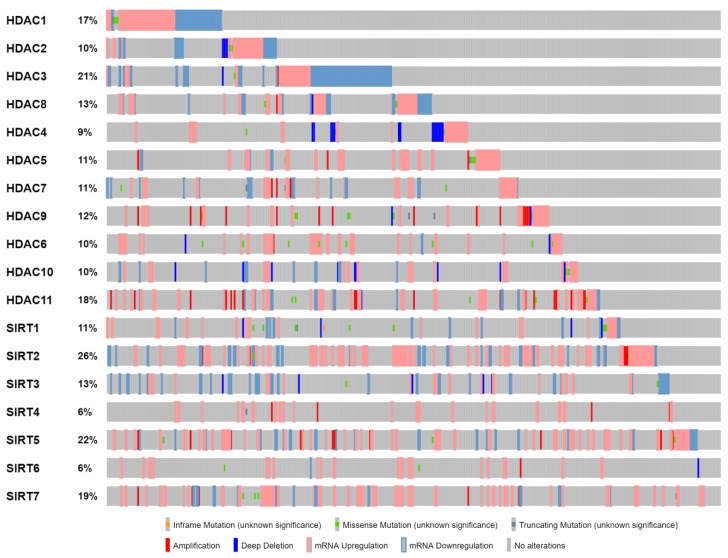
Presentation of genetic alterations being carried by the 18 members of HDAC deacetylase family, using the “cBioPortal” for Cancer Genomics platform that includes 412 bladder cancer (MIBC) patients [28,47], with all information being derived from the publicly available database of TCGA consortium (the Z-score threshold for both mRNA and protein expression was set at 1.5).

A comprehensive analysis of 412 MIBCs that have been thoroughly characterized by multiple platforms of The Cancer Genome Atlas (TCGA) [47] has revealed the patient-specific mutational load and deregulated expression of all 18 HDAC family members [28,165,182,183]. The HDAC-dependent somatic mutation frequency is low, and ranges from 0.2-2.4%, with SIRT1, HDAC9 and HDAC6 presenting the highest measured values among all (Table 1). Remarkably, the positively and negatively charged respective amino acids R and E are those residues that participate with the highest substitution (X to R/E and R/E to X; X: any amino acid) frequencies among all 20 ones being observed in the TCGA mutational MIBC landscapes. This likely suggests the critical roles of HDAC-driven electrostatic interactions in the acetylation/deacetylation processes, and their prominent contribution to bladder cancer initiation, progression and metastasis. Intriguingly, examining the HDAC genetic alterations, including expression changes, heterogeneous patterns are obtained even within a single family member. For example, the ability of HDAC1, 2 and 3 deacetylases to be either upregulated or downregulated in a patient-dependent manner (Figure 3) indicates the decisive role of a tumor’s specific mutational signature to tightly control each HDAC enzyme’s expression and activity. Interestingly, a notable degree of selectivity with respect to the type of genetic change(s) being carried by a particular HDAC family member is also observed in this MIBC patient cohort. As a paradigm, *HDAC4* is subjected to deep deletion more frequently, when compared to the rest of the 17 genes of the family, while *HDAC9* and 11 show the highest percentage of amplification incidents among all examined members (Figure 3). It must be the synergistic aberrant actions of the patient-dependent deregulated HDACs and their target substrates that drive the MIBC pathological phenotype(s).

## 7. Drugging the Bladder Cancer “Acetyl-proteome”: HDAC Inhibitors

**Figure 4:**
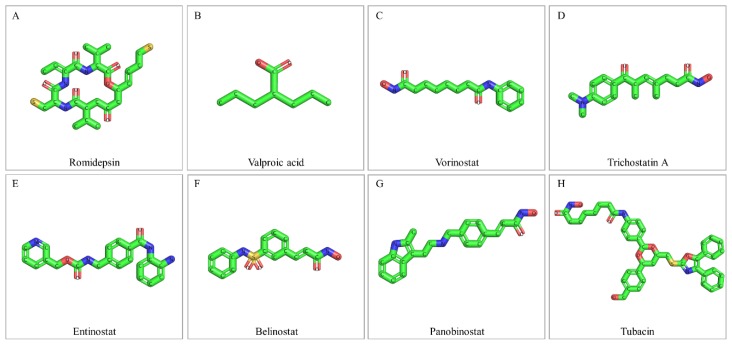
Graphical presentation of the most extensively investigated HDAC inhibitors (HDACis) in bladder cancer. Data were retrieved from the ChEMBL database [184] and visualized via PyMol bioinformatics platform [185].

The epigenetic dysregulation of tumor suppressor genes is considered as an alternative to gene mutations mechanism of cancer pathogenesis. Therefore, epigenetic modification-related enzymes, such as HDACs, can and have already been successfully used as cancer therapeutic targets, since epigenetic control seems to be reversible, and the engaged regulatory proteins can be targeted using small molecules [134]. HDACs are critical mediators of gene expression and their aberrant activity is associated with key oncogenic events [135] (also, see the aforementioned Section 5 and Section 6). Interestingly, high-throughput genome analyses revealed that chromatin regulatory genes are more frequently mutated in bladder cancer, compared to other malignancies [29,39] (also, see the aforementioned Section 2). Therefore, histone deacetylase inhibitors (HDACis) have been employed as promising small-molecule anti-cancer drugs for bladder cancer therapy (Figure 4).

**Figure 5:**
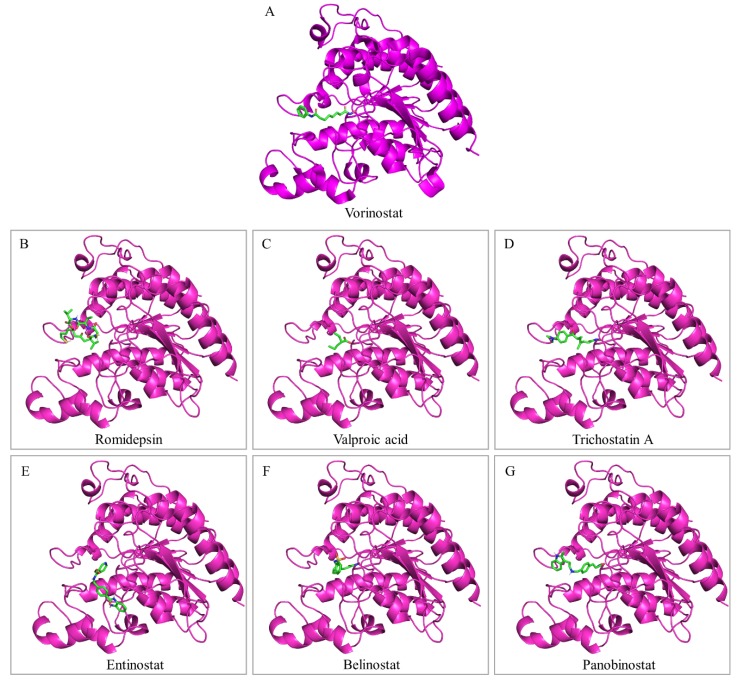
HDACis’ common mechanism of action. Based on (**A**) the experimentally determined structure of human HDAC2 (magenta) in complex with vorinostat (PDB ID: 4LXZ [198]), docking experiments were carried out for the herein reviewed inhibitors, by suitably engaging the AutoDock Vina bioinformatics platform [199]. Docking results of (**B**) romidepsin, (**C**) valproic acid, (**D**) trichostatin A, (**E**) entinostat, (**F**) belinostat and (**G**) panobinostat (green) with HDAC2 indicate that HDACis share a common pattern of structural recognition and molecular mechanism to convey HDAC inhibition. The crystal structure of HDAC2 and the results of docking experiments were visualized via PyMol [185].

HDACis are typically characterized by a molecular structure being comprised of a surface recognition motif (CAP), a zinc-binding group (ZBG) and a linker domain connecting the CAP and ZBG motifs. HDACis’ common mechanism of action is to bind to the catalytic pocket of HDAC and chelate the critical zinc ion required for the catalytic function of the enzyme (Figure 5) [186,187,188,189,190]. HDACis are generally classified by their chemical structure, target selectivity/specificity and inhibitory concentrations which range from nM (nano-molar) to mM (milli-molar) scales [134,186,187]. Regarding their chemical class, HDACis are categorized as: (a) hydroxamic acids (e.g. belinostat, givinostat, panobinostat, trichostatin A, tubacin and vorinostat), (b) benzamides (e.g. entinostat and mocetinostat), (c) short-chain and aromatic fatty acids (e.g. sodium butyrate and valproic acid), and (d) cyclic peptides (e.g. apicidin, romidepsin and trapoxin) [191,192]. Moreover, based on their target specificity, HDACis are typified as: (i) pan-HDACs (e.g. belinostat, panobinostat, sodium butyrate, trichostatin A, valproic acid and vorinostat), (ii) Class I (e.g. entinostat and romidepsin), and (iii) HDAC6 (e.g. tubacin and tubastatin) [134,165,193]. In terms of the required HDACis-concentration range, for the in vitro inhibition of HDAC activities, the hydroxamate and cyclic tetrapeptide compounds serve as the most potent inhibitors, since they function at low micro-molar or even nano-molar scales. In contrast, benzamides inhibit HDACs at micro-molar concentrations, while the short-chain fatty acid compounds usually require milli-molar doses [186,187,194]. A prominent feature of HDACis is their high degree of selectivity for killing cancer cells [195,196]. The exact mechanism remains still unclear, but it seems that normal cells activate repair pathways enabling them to respond more efficiently to damage caused by HDACis; e.g. by reversible cell-cycle arrest and enhanced DNA-damage repair [163,197]. The anti-neoplastic efficacy of HDACis has been explored in a number of human malignancies, but the present review article will discuss only the most widely investigated HDACis in urothelial bladder cancer (Figure 4).

### 7.1. Romidepsin

Romidepsin (FK228, depsipeptide, FR901228) is a Class I-specific HDACi, which belongs to the chemical class of cyclic peptides (Figure 4A and Figure 5B). Under the trade name Istodax (Celgene, NJ, USA), it has been approved by the FDA (Food and Drug Administration) for treatment of advanced cutaneous T-cell lymphoma (CTCL) and peripheral T-cell lymphoma (PTCL) [190,200,201]. Treatment of bladder cancer-cell lines with romidepsin resulted in a dose- and time-dependent growth inhibition, cell-cycle arrest, elevated p21^WAF1^ levels via a p53 independent pathway and apoptosis induction [202]. A time-dependent, tumor-growth suppression was also observed after romidepsin administration in xenograft and orthotopic bladder-cancer in vivo models [202]. Similarly, exposure of bladder cancer-cell lines, with distinct HDAC1 and 2 expression profiles, to romidepsin resulted in significantly reduced proliferation, inhibition of clonogenic growth, disturbed cell-cycle progression, and induction of a mixed apoptotic and necrotic cell death [163]. Interestingly, this study identified romidepsin as the most potent HDACi in bladder cancer-cell lines, as compared to givinostat, entinostat and mocetinostat [163]. These anti-tumor effects of romidepsin can be further enhanced by co-administration of the new compound 2’,3’-di-methoxy-cinnamoyl-azide (DMCA) [203]. In the same direction, a cocktail of romidepsin and JQ1, an inhibitor of bromodomain-containing acetylation-reader proteins, presented synergistic effects in inhibiting cell-cycle progression, suppressing clonogenic growth and inducing caspase-dependent apoptosis [204]. A quantitative proteomics approach indicated that the suppressed cell growth and induced cell death observed in the 5637 bladder cancer-cell line, in response to treatment with romidepsin, vorinostat and trichostatin A, is mediated through modulation of protein expression involved in cell cycle, chromatin modification, apoptosis, autophagy, ROS generation and DNA-damage repair [205]. Oncogenic HRAS activation seems to be tightly associated with the promotion of tumorigenesis in human bladder cancer. Remarkably, treatment of J82 bladder cancer cells that carry oncogenic HRAS with romidepsin leads to increased apoptosis (controlled by ERK-pathway activation and ROS production), as compared to the parental J82 cells that host wild-type *RAS* genes. These observations strongly suggest a potential value of romidepsin in developing anti-cancer therapeutic regimens against RAS-related bladder cancers [206,207,208].

Efficient adenoviral (Ad) infection, which is commonly used as a delivery system in gene-therapy protocols, requires the presence of coxsackievirus and adenovirus type-5 receptor (CAR). CAR, typically, is a cell-adhesion molecule linked to cell motility and tumor invasiveness [209], and its decreased expression in bladder cancer is associated with cancer-cell growth [210,211]. Romidepsin administration in a panel of bladder cancer-cell lines significantly induced CAR expression and acetylated Histone H3, and resulted in marked enhancement of transgene expression after adenoviral infection. Hence, romidepsin and Ad-CMV-p53 combination proved capable to cause a significant tumor inhibition in vitro and in vivo, thus suggesting that a pre-treatment step of tumor cells with romidepsin may increase the efficiency of adenoviral gene-therapy vectors [212].

### 7.2. Valproic Acid

Valproic acid (VPA) is a pan-HDACi of the short-chain fatty acids chemical class (Figure 4B and Figure 5C) and an FDA-approved treatment option for epilepsy and bipolar disorder. Although it is not FDA-indicated for the treatment of cancer, valproic acid has been extensively studied for its potential therapeutic use in various malignancies, including bladder cancer. Valproic acid administration in T24, TCCSUP, HT1376 and RT4 bladder cancer-cell lines caused a dose-dependent increase in acetylated Histone H3 and p21^WAF1^ expression, and a decrease in invasion rate (except RT4), but had no effect in migration capacity. Moreover, valproic acid significantly inhibited tumor growth of T24 xenograft models [213]. Exposure of HT1376 and 5637 bladder cancer cells to valproic acid resulted in a dose-dependent inhibition of cell survival and increased sub-G1 cell population, probably due to elevated levels of p21^WAF1^ protein expression [164]. Time- and dose-dependent growth-inhibiting actions of valproic acid against RT4, TCCSUP, UMUC3 and RT112 bladder cancer-cell lines have also been observed. Valproic acid enhanced the acetylation of Histones H3 and 4, decreased HDAC3 and 4 protein expression, upregulated p27^KIP1^ and reduced the cell-cycle regulators Cdk1, 2 and 4, and Cyclin B, D1 and E [214]. Valproic acid significantly blocked adhesion of TCCSUP and RT112 bladder cancer cells to collagen, under flow conditions, by altering their integrin expression pattern, therefore suggesting a promising option for the prevention of tumor dissemination [215].

XPC is a DNA-damage recognition protein required for the initiation of the nucleotide-excision repair (NER) process and reduced levels of XPC protein have been reported in tumors derived from bladder cancer patients. Valproic acid administration in T24 and 5637 bladder cancer cells induced transcription of *XPC* gene, while prior treatment of these cells with valproic acid enhanced cisplatin-induced activation of caspase-3 [173]. In another study, exposure of UMUC3 and T24 bladder cancer-cell lines to valproic acid and vorinostat led to decreased proliferation and increased expression of thrombospondin-1, a natural inhibitor of angiogenesis [216]. The effectiveness of valproic acid was greatly enhanced in human bladder cancer cells, when it was combined with the DNA-damaging agent mitomycin C, likely due to the augmented cytotoxic activity of mitomycin C in response to chromatin-structure ”relaxation“ caused by valproic acid [217]. Similarly, elevated bladder cancer cell-growth inhibition was achieved when valproic acid was combined with mitomycin C, cisplatin and adriamycin, in vitro and in vivo [218]. Notably, valproic acid has been recently demonstrated to reduce the resistance of bladder cancer cells to the mTOR inhibitor temsirolimus, by suppressing, among others, the Cdk2/Cyclin A axis [219]. Interestingly, valproic acid has also been shown to upregulate CAR in T24 bladder cancer cells, thus supporting the idea of valproic acid addition to adenoviral-mediated cancer gene-therapy protocols [220]. Finally, regarding the pharmacokinetics and whole-body distribution of valproic acid, an in vivo study revealed its relatively long half-life time, very low brain uptake, high heart and liver uptake, and its accumulation in the bladder [221].

### 7.3. Vorinostat

Vorinostat (SAHA: suberanilohydroxamic acid) is a pan-HDACi that belongs to the class of hydroxamic acids (Figure 4C and Figure 5A) and under the trade name Zolinza (Merck & Co., Inc., NJ, USA) was the first HDAC inhibitor approved by the FDA for treatment of CTCL [190,222]. Vorinostat administration in T24 bladder cancer cells resulted in cell proliferation inhibition, accumulation of acetylated Histones H3 and 4, and elevated expression levels of p21^WAF1^ mRNA and protein, an induction that may be regulated, at least in part, by histone acetylation in chromatin associated with the promoter and coding regions of *p21^WAF1^* gene [223]. Microarray studies in T24 bladder cancer cells after vorinostat and trichostatin A treatment demonstrated an upregulation in gene expression for *p21^WAF1^*, *Hep27*, *Histone H2B*, *TRPM2*, *Gelsolin*, *α-Tubulin*, *Glutaredoxin* and *Metallothionein 1L*, and a repression in *Thymidylate Synthase*, *CTP synthase*, *APRIL* and *TRP* gene expression [224]. In another study, exposure of urothelial cancer-cell lines to vorinostat resulted in a dose-dependent decline of cell viability, a prominent G2/M arrest, a p21^WAF1^ upregulation, a Thymidylate Synthase downregulation and an induction of Caspase 3/7 activities [171]. In bladder cancer cells, elevated Nuclear Receptor Co-repressor 1 (NCOR1) expression generates an epigenetic lesion that is associated with increased cellular invasiveness and attenuated anti-proliferative receptor responses. Targeting the epigenetic lesion with vorinostat significantly enhanced anti-proliferative receptor sensitivity, in cell lines with elevated NCOR1, and significantly altered genome-wide transcriptional responses [225]. Finally, in a recent study, the examination of 59 urothelial cancer patients revealed higher *Notch3* contents in bladder tumor tissues, as compared to control (non-cancerous) samples. This Notch3 over-expression was associated with poor prognosis and shorter overall patient survival. Treatment of T24 bladder cancer cells with vorinostat upregulated Notch3 protein-acetylation levels, decreased Notch3 expression, reduced proliferation and induced cell-cycle arrest, thus indicating the integration of vorinostat in bladder cancer therapeutic schemes [226].

### 7.4. Trichostatin A

Trichostatin A (TSA) is a pan-HDACi that belongs to the chemical class of hydroxamic acids (Figure 4D and Figure 5D). TSA administration in a panel of bladder cancer-cell lines led to a concentration-dependent growth inhibition, elevated *p21^WAF1^* transcript contents, Plakoglobin upregulation, and cell death [227]. Similar results of cell growth inhibition, increased *p21^WAF1^* mRNA expression, G1 cell-cycle arrest and apoptotic cell death were obtained in BIU-87 cells, in response to treatment with TSA [228]. Cao et al. (2015) revealed that the TSA-induced apoptosis in T24 bladder cancer cells is related to TRPM2 (Transient Receptor Potential Cation Channel, Subfamily M, Member 2) upregulation, which is caused by increased H3K9Ac enrichment within the *TRPM2* promoter [229]. In another study by Wang et al. (2017), TSA administration initially activated the intrinsic apoptotic pathway associated with phosphorylated Akt inhibition and mitochondrial membrane-potential loss, followed by downregulation of the Sp1-Survivin pathway at the late phase of treatment [230]. In vivo TSA administration in mice bearing EJ and UMUC3 bladder cancer xenografts led to a 70% tumor growth suppression and absence of detectable toxicity [227]. Using T24 bladder cancer cells, Ou et al. (2007) showed that the TSA-induced histone hyper-acetylation is associated with a significant decrease in global gene methylation. Interestingly, TSA could restore the transcription of certain methylated genes, such as *E-cadherin* and *RARβ2*, thus suggesting a reversible cross-talk between histone acetylation and DNA demethylation processes [231]. As it has been previously reported by Yoon et al. (2011), TSA proved able to synergistically enhance the anti-tumor effects of cisplatin and to re-sensitize the T24R2 cisplatin-resistant bladder cancer cells, by potentiating cisplatin-mediated S- and G2/M-phase cell-cycle arrest and Caspase-dependent apoptosis [232]. TSA could also synergistically enhance the gemcitabine-induced cell-cycle arrest, activation of Caspase-dependent apoptosis and suppression of NFκB and Akt signaling [233]. Furthermore, TSA was capable to decrease migration ability and phosphorylated AKT (Ser^473^) expression in bladder cancer cells, and to inhibit TGIF (TG-Interacting Factor), whose over-expression is correlated with poor prognosis and chemoresistance to gemcitabine [234]. These data suggest the use of TSA specifically, and HDACis in general, as combination agents to overcome resistance and enhance the anti-tumor actions of cisplatin and gemcitabine in patients with advanced bladder cancer. Accordingly, pre-treatment with TSA, against TRAIL-resistant T24 bladder cancer cells, followed by incubation with TRAIL, resulted in a marked increase of TRAIL-induced apoptosis, due to upregulated TRAIL-R2 expression, thus indicating the combination of TSA and TRAIL as a promising alternative treatment for urothelial bladder cancer therapy [235]. As mentioned above, CAR expression is commonly downregulated in bladder tissue tumors [210,211]. TSA administration in CAR-negative bladder cancer-cell lines caused a significant elevation of CAR expression, leading to enhanced adenoviral-mediated gene transfer [236]. Notably, El-Zawahry et al. (2006) verified the increased CAR contents in T24 bladder cancer cells, in response to TSA treatment [237]. Most importantly, the TSA-induced CAR upregulation augmented the efficacy of adenovirus-expressing TRAIL (AdTRAIL) to activate apoptosis [235,237]. Interestingly, TSA was presented as able to generate a strong pro-apoptotic phenotype, by reducing the expression of cFLIP and Bcl-X_L_ anti-apoptotic proteins [237].

### 7.5. Entinostat

Entinostat (MS275) is a Class I HDACi, which belongs to the chemical group of benzamides (Figure 4E and Figure 5E). In a study of Qu et al. (2010), entinostat and TSA proved to significantly inhibit the growth of T24 bladder cancer cells, by inducing cell-cycle arrest in G_0_/G_1_-phase and activating apoptosis. They could significantly increase the acetylation contents of Histone H3, induce *p21^WAF1^* mRNA expression and downregulate *Cyclin A* mRNA levels [238]. In another study that mainly focused on adhesion-related proteins, the entinostat, TSA, apicidin and valproic acid were applied in a panel of bladder cancer-cell lines. All four HDACis caused a dose-dependent inhibition of cell growth, upregulation of γ-Catenin in highly invasive lines, and increased Gelsolin, but decreased Desmoglin protein expression in poorly and moderately invasive lines. Entinostat-treated cells were also characterized by an upregulation of α-Catenin in highly and moderately invasive lines, and of E-cadherin in cell lines with moderate and poor invasive potential, thus suggesting the efficacy of entinostat to suppress tumor growth and invasion [239]. Exposure of T24 bladder cancer cells to entinostat and vorinostat led to an EMT reversal (MET; mesenchymal to epithelial transition), and epithelial-differentiation restoration, by recovering E-cadherin and ErbB3 expression, and increasing anoikis [240]. Lee et al. (2016) suggested that Kaposi’s sarcoma-associated herpesvirus (KSHV), which is detected at high frequencies in patient-derived bladder cancer tissues, may be related to drug resistance of bladder cancer cells infected with KSHV. Treatment of KSHV-infected TCCSUP bladder cancer cells with entinostat and vorinostat proved capable to significantly increase the cisplatin-induced cytotoxicity, therefore indicating a potential therapeutic role of HDACis against drug-resistant bladder cancer cells being originated from KSHV infection [241].

### 7.6. Belinostat

Belinostat (PXD101) is a pan-HDACi, which belongs to the class of hydroxamic acids (Figure 4F and Figure 5F) and under the trade name Beleodaq (Spectrum Pharmaceuticals, CA, USA) has been recently approved by the FDA for treatment of relapsed or refractory PTCL [242,243]. The first study of belinostat administration in a panel of human urinary bladder cancer-cell lines (5637, T24, J82 and RT4) unveiled its capacity to induce cell growth inhibition and cell-cycle arrest. Belinostat could decrease bladder tumor growth in a HRAS transgenic-mouse model, with lack of detectable toxicity, and also induce *p21^WAF1^* expression [244]. A new method of nanoparticle-mediated belinostat release, with nanoparticle’s surface having been modified with the novel cell-penetrating polymer PGON (polycationic PGON likely helps the nanoparticles to adhere on the bladder wall and open cellular junctions), demonstrated the greatly enhanced cell penetration in the urothelium of mouse bladder and human ureter, and higher load, of belinostat, as compared to chitosan-functionalized nanoparticles. Remarkably, these belinostat-encapsulated nanoparticles induced prolonged HDAC inhibition in vitro and in vivo, and suppressed bladder tumor growth in a xenograft model, therefore reducing the need for frequent belinostat dosing [245]. Most importantly, a limited phase-II study of belinostat administered in combination with carboplatin and/or paclitaxel in patients with transitional carcinoma of the bladder has shown promise, although the final results are awaited [246].

### 7.7. Panobinostat

Panobinostat (LBH589) is a pan-HDACi, which belongs to the class of hydroxamic acids (Figure 4G and Figure 5G) and under the trade name Farydak (Novartis, Basel, Switzerland) has been approved by the FDA as a combination therapy agent, along with bortezomib and dexamethasone, in patients with recurrent multiple myeloma (MM), who have received at least two prior treatment regimens, including bortezomib and an immunomodulatory agent [247]. Panobinostat was found to act as an efficient radio-sensitizer in RT112 bladder cancer cells, and was associated with the downregulation of the homologous recombination-related proteins MRE11, NBS1 and RAD51 [248]. The post-transcriptional downregulation of MRE11 and the production of a truncated MRE11 form, which is degraded by the proteasome, are mediated through cIAP2 function [249]. Interestingly, panobinostat exhibited a comparatively greater radio-sensitizing effect in RT112 bladder cancer cells that were knocked down for Ku80, and since muscle-invasive bladder tumors contain reduced Ku-DNA binding activities, panobinostat could serve as an important radio-sensitizer in bladder cancer [248].

### 7.8. Tubacin

Tubacin (Tubulin acetylation inducer) is a highly potent and selective HDAC6 inhibitor that belongs to the class of hydroxamic acids (Figure 4H) and is characterized by a stronger activity than tubastatin A and ST-80, the other two HDAC6-specific inhibitors hitherto available [174,250]. HDAC6 has been previously shown to act synergistically with SIRT2, in promoting bladder cancer-cell migration and invasion, by targeting the cytoskeletal protein Cortactin in the cell line BLX211. Tubacin administration at micro-molar concentrations, combined with the siRNA-mediated knockout of SIRT2, caused the suppression of cell migration and invasion, thus indicating tubacin as a promising therapeutic agent for bladder cancer therapy [178]. In contrast, HDAC6-specific inhibition through the HDACis tubacin, tubastatin A and ST-80 across a panel of urothelial cancer-cell lines, including bladder cancer ones, revealed only moderate efficacies in the induction of cell-cycle arrest or cell viability reduction, and exerted only minor effects on migration and invasion, hence suggesting that growth and survival of urothelial cancer cells may not critically depend on HDAC6 function [174]. However, in a recent study, HDAC6 inhibition in RT112 bladder cancer cells by tubacin resulted in induction of apoptosis and proliferation arrest, which was associated with the downregulation of FGFR3, MYC and Cyclin D1, and DNA-damage induction. Notably, tubacin was well-tolerated and could significantly impede RT112 tumor growth in xenoplant assays [250].

Conclusively, although HDAC inhibition seems to serve as a promising strategy for bladder cancer therapy, accumulated evidences suggest that for maximizing the anti-tumor effects of HDACis, these may not be used as monotherapy agents, but in combination with other already established drugs and therapeutic schemes, including radiation.

## 8. Clinical Trials

Hitherto, four different HDACis have been approved by the FDA for cancer therapy. In 2006, vorinostat was the first HDACi to be approved for the treatment of progressive, persistent or recurrent CTCL, following two systemic therapies [222]. Similarly, romidepsin was licensed in 2009 for the treatment of CTCL patients, who have received at least one prior systemic therapy [251]. The use of belinostat against relapsed or refractory PTCL was approved by the FDA in 2014 [243], while more recently, in 2015, panobinostat, in combination with bortezomib and dexamethasone, was also approved for the treatment of MM patients following at least two prior regimens [252].

The principle in using HDACis as, small-molecule, anti-cancer drugs is their capacity to increase core histones’ acetylation, hence strongly triggering an open-chromatin state, and consequently the high accessibility of DNA-targeting agents. In this regard, the mode-of-action of HDACis mainly includes cell-cycle arrest, terminal differentiation and cell death in transformed cancer cells, but not in normal cells, which are presented as either insensitive or capable to effectively overcome the drug-induced stress. Additionally, HDACis can also exert their tumor-suppressing activities by inhibiting angiogenesis and/or modulating immune responses [65,253,254]. Numerous different clinical trials (423 as of January 2019; [255]) of HDACis for the therapeutic treatment of human malignancies have been completed, or are ongoing (recruiting, active-not-recruiting, enrolling and not-yet-recruiting status). Considering the limited therapeutic effect of HDACis as monotherapy in several solid tumors, the majority of current trials are focused on the synergistic effects of HDACis in combination with other anti-cancer agents/drugs, or radiotherapy protocols [254,256].

Combination schemes of HDACis with agents targeting DNA damage-response (DDR) pathways or topoisomerase inhibitors have been reported to effectively increase DNA damage, cell-cycle arrest and death of cancer cells, as compared to monotherapy. Interestingly, HDACis have been documented to induce DNA double-strand breaks (DSBs), as well as to attenuate DDR, through reduction of ATM and p53 activities, specifically in cancer cells [257,258]. Similarly, ongoing clinical trials are currently evaluating the effectiveness and safety of (a) the combination of olaparib (a PARP inhibitor) with vorinostat in relapsed/refractory and/or metastatic breast cancer (NCT03742245), as well as (b) the high-dose cocktail chemotherapy (vorinostat, gemcitabine, busulfan and melphalan, either with or without rituximab) in patients with non-Hodgkin’s or Hodgkin’s lymphoma, who are receiving an autologous stem-cell transplant (NCT03259503). Remarkably, a completed phase-I clinical study (NCT00246103), combining valproic acid and epirubicin (a Topoisomerase-II inhibitor) unveiled the well-tolerated character of the administered scheme, and the advanced clinical response rate of patients with solid tumors [259]. In the same direction, several trials are evaluating the clinical behavior of HDACis and doxorubicin (a Topoisomerase-II inhibitor) combinations. In this regard, a phase-I/II trial of belinostat with doxorubicin in soft tissue sarcoma patients (NCT00878800) demonstrated its superior median time to disease progression related to doxorubicin monotherapy [260]. On the other hand, a completed phase-I trial of vorinostat combined with doxorubicin (NCT00331955) showed poor clinical benefits [261].

The ability of HDACis to attenuate DDR leads to an enhanced radio-sensitivity of cancer cells, with favorable results having been reported by the combination of HDACis with radiotherapy [262]. In the clinical setting, phase-I trials have proved that HDACis can be safely administered with radiotherapy, including (a) vorinostat in patients with advanced or metastatic NSCLC (NCT00821951; [263]) and non-metastatic pancreatic cancer (NCT00983268; [264]), and (b) panobinostat for the treatment of prostate, esophageal and head/neck cancers (NCT00670553). Furthermore, a phase-II single group trial of valproic acid with temozolomide and external beam radiation for the treatment of glioblastoma (NCT00302159) showed that the combination scheme was well-tolerated by the patients, and also presented an improved therapeutic outcome [265].

Focusing on platinum-based chemotherapy, phase-I trials of belinostat or vorinostat, in combination with carboplatin and paclitaxel, in patients with solid tumors, underscored the well-tolerance of the scheme, along with notable benefits in patients’ response rates [266,267]. In accordance, a phase-II clinical trial of carboplatin and paclitaxel, with or without vorinostat, in advanced NSCLC demonstrated the improved response and survival outcome of the patients being treated with carboplatin-paclitaxel-vorinostat, as compared to the carboplatin-paclitaxel only treated cohort (NCT01413750; [268]). Unfortunately, the subsequent phase-II/III trial (NCT00473889) in advanced NSCLC (stages IIIB/IV) did not confirm the clinical benefit of vorinostat co-administration.

Additionally, numerous phase-I trials have also focused on the combination of HDACis with Receptor Tyrosine Kinase (RTK)-targeted agents, including sorafenib (a multi-kinase inhibitor of MAPK pathway) and gefitinib (an EGFR inhibitor). Interestingly, an ongoing phase-I/II single group trial (NCT00918333) on the combination of everolimus (an mTOR inhibitor) with panobinostat for the treatment of recurrent MM, non-Hodgkin’s and Hodgkin’s lymphoma has already shown the absence of severe adverse effects. However, a phase-Ib/IIa trial on the combination of trastuzumab (a HER2-targeted monoclonal antibody, blocking the downstream PI3K-AKT pathway) with panobinostat in HER2-positive metastatic breast cancer did not prove a clinical benefit for the patients (NCT00567879).

Immune-checkpoint inhibitors represent one of the most widely examined categories of anti-cancer agents nowadays and are already approved by the FDA for cancer therapy. Several studies have pointed out the ability of HDACis to regulate immune responses, and their combination with immune-checkpoint inhibitors has been presented to improve clinical outcomes [269,270]. In this regard, the HDACi entinostat has been reported to target myeloid-derived suppressor cells, and to improve the anti-tumor efficacy of PD-1 blockage in lung, renal and breast carcinomas [271,272]. Among several ongoing trials of immune-checkpoint inhibitors in combination with HDACis, pembrolizumab (anti-PD-1) have been evaluated along with (a) vorinostat against advanced renal and urinary bladder carcinomas (NCT02619253) and (b) entinostat for the treatment of metastatic melanoma of the eye (NCT02697630), while nivolumab (anti-PD-1), in combination with azacytidine and entinostat, was evaluated for the treatment of metastatic NSCLC patients (NCT01928576). Finally, a phase-I/II single group study of entinostat with aldesleukin (recombinant Interleukin-2) in metastatic kidney cancer (NCT01038778) highlighted the favorable response rate and survival outcome of the patients [273].

### Urothelial Bladder Carcinoma

Focusing on urothelial bladder carcinoma (Table 2), vorinostat has been evaluated as a monotherapy agent for the treatment of patients with locally recurrent or metastatic cancer of the urothelium, in a phase-II single group study (NCT00363883). However, the clinical benefit on patient outcome was limited, while serious adverse effects were highlighted in 43% of the patients, thus resulting in termination of the trial. Moreover, administration of mocetinostat as a single agent in patients with inactivating mutations or deletions in the *CREBBP* and/or *EP300* acetyltransferase genes, previously treated for locally advanced or metastatic urothelial bladder carcinoma, has been studied in a completed phase-II trial (NCT02236195). This trial represents the first genomic-based approach for the identification of patients that are suitable (or more likely to benefit) for treatment with HDACis; however, severe toxicities were observed, compromising the overall clinical therapeutic success [274]. On the other hand, the combination of belinostat with carboplatin or paclitaxel in a phase-I/II single arm study (NCT00421889), evaluating safety, pharmacodynamics and efficacy, revealed complete or partial responses in four out of 15 bladder cancer patients enrolled. Finally, vorinostat is currently being evaluated, in combination with pembrolizumab, in an ongoing phase-I/Ib study, against advanced renal or urothelial cell carcinoma (NCT02619253).

## 9. Conclusions

The cellular “acetyl-proteome” and especially “acetyl-chromatin” have emerged as major druggable landscapes for human malignancies. Targeting the plethora of molecular “writers” (HATs) and “erasers” (HDACs), along with their cognate substrates, opens a new therapeutic window for the disease. However, the presumable tissue-specific expression and/or activity of HAT/HDAC family members necessitate their cancer cell-dependent molecular profiling before treatment. In the same direction, given the heterogeneity of a solid tumor, different sub-populations may express distinct *HAT*/*HDAC* gene combinations with variable oncogenic activities. To further complicate things, there could be more than one family member able to modulate the “acetyl-load” of a given target, while many substrates might be specifically targeted by only one (HAT/HDAC) member. Moreover, presumable operation of redundant responses, during which changes in one protein’s acetylation levels are compensated by critical changes in a different protein, most likely stiffen and worsen our comprehension, and view of the system’s functionality. These are most likely some of the reasons justifying the “failure” of HDACi-mediated monotherapy to meet its promise, since only combination schemes are presented beneficial for the patients. A new generation of HAT/HDAC-specific inhibitors and novel cocktail-drug/radiation protocols are expected to overcome the existing limitations, and offer the desired therapeutic success for several human malignancies.

In recent years, HDACs have emerged as key players to cellular homeostasis. Deregulation of these enzymes ultimately leads to several malignancies, including bladder cancer, by various mechanisms. This review brings together important information about HDACs and their specific inhibitors, with some of them already being tested in clinical trials, demonstrating potential for treating urothelial cancers. The knowledge gathered in this study regarding HDACis, provides insights necessary for directing future research in this field.

## Figures and Tables

**Table 1:** Collection of mutations detected in the 18 HDAC family members, using a cohort of 412 patients being affected by bladder cancer (MIBC). All data were provided by the TCGA consortium [28,47].

The Cancer Genome Atlas (TCGA)—Bladder Cancer			
Family Member	Protein Change	Mutation Type	Somatic Mutation Frequency
HDAC1	Q364*, M64I, L276I, Y204S	N, M, M, M	1.0%
HDAC2	E204K, H180R, R446K	M, M, M	0.7%
HDAC3	F8L	M	0.2%
HDAC8	E23K, R361Q, V175M	M, M, M	0.7%
HDAC4	E587Q, D54N, S290F	M, M, M	0.7%
HDAC5	Q468*, Q88L, E620K, E591Q, E614K, S886C	N, M, M, M, M, M	1.2%
HDAC7	D937N, G36Afs*11, M828I, P320Rfs*3	M, FS del, M, FS del	1.0%
HDAC9	H935L, Q710H, E49*, S1038F, S3T, G1022Afs*22, G133V, L411V, S522F	M, M, N, M, M, FS del, M, M, M	2.2%
HDAC6	R236L, H1115R, G344E, G1014W, E333Q, E432Q, E562D, W705*, D1128H, R480C, T260A, V874F	M, M, M, M, M, M, M, N, M, M, M, M	2.2%
HDAC10	S545C, A434V, R411H	M, M, M	0.7%
HDAC11	R206H, N47H, D282H, V19M, H35Q	M, M, M, M, M	1.2%
SIRT1	H471Y, A139del, Y650D, Q632H, E230K, D197H, S550*, E609A, G618E, E164K	M, IF del, M, M, M, M, N, M, M, M	2.4%
SIRT2	R346Q, R163C	M, M	0.5%
SIRT3	H217R, G161R	M, M	0.5%
SIRT4	S208*, L227V, S302C	N, M, M	0.2%
SIRT5	R44Q, L246V, E125K	M, M, M	0.7%
SIRT6	E139K, E131Q	M, M	0.5%
SIRT7	A258Cfs*31, E203K, E203K, R289W	FS ins, M, M, M	1.0%

*: Stop codon; N: Nonsense; M: Missense; FS: Frameshift; del: Deletion; IF: Inframe; ins: Insertion.

**Table 2:** Clinical trials of HDACis in urothelial bladder cancer (BlCa: Bladder Cancer) [255].

HDACi Agent	Combined Agent	Phase	Status	Patients	Outcome	Identifier
Vorinostat	-	Phase-II	Terminated	Locally recurrent, or metastatic cancer of the urothelium	Limited clinical benefit; Serious adverse effects (43%)	NCT00363883
Mocetinostat	-	Phase-II	Completed	BlCa patients with inactivating mutations or deletions in the *CREBBP* and/or *EP300* acetyltransferase genes	Severe toxicities; Limited clinical efficacy	NCT02236195
Belinostat	Carboplatin or paclitaxel	Phase-I/II	Completed	BlCa patients	Complete or partial responses in 4 out of 15 BlCa patients enrolled	NCT00421889
Vorinostat	Pembrolizumab	Phase-I/Ib	Ongoing	Advanced urothelial cell carcinoma		NCT02619253

## Data Availability

The datasets and materials supporting the conclusions of this article are included within the article.
